# P72 Research experience across the paediatric rheumatology multi-disciplinary team

**DOI:** 10.1093/rap/rkac067.072

**Published:** 2022-09-28

**Authors:** Kirsty McLellan, Charlene Foley, Victoria Harbottle, Heather Smee, Ethan S Sen

**Affiliations:** Great Ormond Street Hospital, London, United Kingdom; Great Ormond Street Hospital, London, United Kingdom; Great North Children's Hospital, Newcastle, United Kingdom; Bristol Children's Hospital, Bristol, United Kingdom; Great North Children's Hospital, Newcastle, United Kingdom; Faculty of Medical Sciences, Newcastle University, Newcastle, United Kingdom

## Abstract

**Introduction/Background:**

Embedding research into clinical practice has many benefits, with research-active healthcare settings reporting better clinical outcomes and improved staff recruitment and retention. This is recognised by the NHS who aim to ‘build the capacity and capability of our current and future workforce to embrace and actively engage with research’. In spite of this, clinical academic capacity across the NHS remains challenging; the number of consultants working in clinical academia has declined in recent years and there is concern about lack of academic progression for non-medical professions. Reported barriers include clinical pressures and lack of dedicated time, individual skill and confidence.

**Description/Method:**

Our aim was to gather information about research exposure across the Paediatric Rheumatology multidisciplinary team (MDT), including paediatric trainees (rheumatology grid, rheumatology spin and level 2 trainees), clinical nurse specialists (CNS), advanced nurse practitioners (ANPs) and Allied Health Professionals (AHPs).

We initially sought to identify if trainees were receiving adequate research opportunities during their training. A pilot questionnaire was distributed, and results collated and presented at the Spring Clinical Studies Group (CSG) annual meeting. Feedback was received from both questionnaire respondents and the CSG. Following this, we modified and broadened the scope of the questionnaire to include the Paediatric Rheumatology MDT, with the aim of comparing experiences across the MDT. This was developed using an online survey platform with the link distributed via email and messaging groups for trainees, AHPs and CNSs.

The aims of the modified questionnaire were to;

1. Understand the current research experience across the paediatric rheumatology MDT and identify barriers and ways to support participation in research.

2. Identify if individuals wanted more exposure to research and what specific research skills they would like to develop.

**Discussion/Results:**

There were 34 respondents: 14 (41%) paediatric trainees (7 grid, 2 spin, 2 post-CCT fellows, 3 level-2 trainees), 14 (41%) CNS, 4 (12%) AHPs and 2(6%) ANPs.

Across the MDT, 19 respondents (56%) agreed they had adequate opportunity to be involved in research, of which 7 (21%) strongly agreed. In terms of research exposure, 22 (65%) have undertaken postgraduate degrees, 5 (15%) PhD, 9 (26%) MSc, 5 (15%) diploma and 6 (18%) postgraduate certificate. Eight-respondents (24%) had taken time out to develop research skills.

**Research experience:**

18 respondents (53%) have been on the delegation-log for clinical trials. 20 (59%) have contributed to data collection for National Registries. 23 (68%) have given a poster/oral presentation at national/international conferences and 15 (44%) have published in peer-reviewed journals - the majority trainees (n = 11,73%).

**Research training:**

51% report adequate training in critical appraisal, 48% in literature review and 40% in consent; fewer reported adequate training in designing research projects (21%), ethics applications (21%) and statistical analysis (23%).

Further training would be desirable in:

• designing research projects (68%);

• discussing research with patients (65%);

• statistical analysis (49%);

• critical appraisal (40%);

• literature search (37%).

**Future research involvement:**

94% would like more opportunities to be involved in research. In the future, 63% would like allocated research time, 6% to be mainly academic (1 CNS, 1 trainee) and 12% to be full-time clinical (1 spin, 1 level-2 trainee, 2 CNS).

**Barriers to research:**

Free text answers were used to gather information and common themes identified included lack of:

• time/heavy clinical commitment (60%);

• supervision/support from seniors (9%);

• local resources (9%);

• research funding (9%);

• awareness of projects (12%);

• research skills (9%).

**Encourage participation in research:**

Common suggestions included:

• protected research time during training/career (35%);

• further research training (21%);

• earlier awareness of projects/trials (18%);

• Increased support from more experienced colleagues (15%);

• improved collaboration/networking (12%).

**Key learning points/Conclusion:**

This survey provides an interesting insight into the research experience throughout the Paediatric Rheumatology MDT.

It is encouraging that within the Rheumatology MDT 56% of respondents reported that they have had adequate opportunities to be involved in research and the majority have presented or published their research. However, 94% have reported they would like more opportunities to be involved in research. Lack of time was reported as the most common barrier to research involvement; this finding is consistent with the British Society for Rheumatology’s 2019 ‘Paediatric State of Play’ report. Additionally, significant numbers report they would like further training in research skills. Academic writing for publication was noted as a particular area of concern for nurses and AHPs.

We advocate for further research opportunities throughout the MDT. For Rheumatology trainees, it was suggested that research skills be incorporated into the curriculum, with dedicated time allocated to gain experience and contribute to research. All members of the MDT may benefit from research skills training courses, although this would need to be carefully considered, given lack of time and resources were common barriers reported by respondents. Several professionals reported lack of support or supervision from seniors and suggested the benefit of mentoring networks.

A weakness of this study is the relatively low number of respondents; the survey remains open and we intend to collect further data to maximise representation across the MDT. Additionally, there is potential bias, with individuals with a research interest possibly more likely to contribute, meaning research experience may be over-represented by this sample of the MDT. Also, representation from AHPs was limited compared to paediatric trainees and CNS.

Further work is needed to understand research experience across the MDT, however this initial survey is a valuable first step in encouraging discussion of MDT participation in research.

